# Hippocampal hyperphosphorylated tau-induced deficiency is rescued by L-type calcium channel blockade

**DOI:** 10.1093/braincomms/fcae096

**Published:** 2024-03-20

**Authors:** Chelsea A Crossley, Tamunotonye Omoluabi, Sarah E Torraville, Sarah Duraid, Aida Maziar, Zia Hasan, Vishaal Rajani, Kanae Ando, Johannes W Hell, Qi Yuan

**Affiliations:** Biomedical Sciences, Faculty of Medicine, Memorial University of Newfoundland, St. John’s, NL A1B 3V6, Canada; Biomedical Sciences, Faculty of Medicine, Memorial University of Newfoundland, St. John’s, NL A1B 3V6, Canada; Biomedical Sciences, Faculty of Medicine, Memorial University of Newfoundland, St. John’s, NL A1B 3V6, Canada; Biomedical Sciences, Faculty of Medicine, Memorial University of Newfoundland, St. John’s, NL A1B 3V6, Canada; Biomedical Sciences, Faculty of Medicine, Memorial University of Newfoundland, St. John’s, NL A1B 3V6, Canada; Biomedical Sciences, Faculty of Medicine, Memorial University of Newfoundland, St. John’s, NL A1B 3V6, Canada; Biomedical Sciences, Faculty of Medicine, Memorial University of Newfoundland, St. John’s, NL A1B 3V6, Canada; Department of Biological Sciences, School of Science, Tokyo Metropolitan University, Tokyo, 192-0397, Japan; Department of Pharmacology, School of Medicine, University of California at Davis, Davis, CA 95616, USA; Biomedical Sciences, Faculty of Medicine, Memorial University of Newfoundland, St. John’s, NL A1B 3V6, Canada

**Keywords:** tau, hippocampus, Alzheimer’s disease, spatial learning, L-type calcium channel

## Abstract

Aging and Alzheimer’s disease are associated with chronic elevations in neuronal calcium influx *via* L-type calcium channels. The hippocampus, a primary memory encoding structure in the brain, is more vulnerable to calcium dysregulation in Alzheimer’s disease. Recent research has suggested a link between L-type calcium channels and tau hyperphosphorylation. However, the precise mechanism of L-type calcium channel-mediated tau toxicity is not understood. In this study, we seeded a human tau pseudophosphorylated at 14 amino acid sites in rat hippocampal cornu ammonis 1 region to mimic soluble pretangle tau. Impaired spatial learning was observed in human tau pseudophosphorylated at 14 amino acid sites-infused rats as early as 1–3 months and worsened at 9–10 months post-infusion. Rats infused with wild-type human tau exhibited milder behavioural deficiency only at 9–10 months post-infusion. No tangles or plaques were observed in all time points examined in both human tau pseudophosphorylated at 14 amino acid sites and human tau-infused brains. However, human tau pseudophosphorylated at 14 amino acid sites-infused hippocampus exhibited a higher amount of tau phosphorylation at S262 and S356 than the human tau-infused rats at 3 months post-infusion, paralleling the behavioural deficiency observed in human tau pseudophosphorylated at 14 amino acid sites-infused rats. Neuroinflammation indexed by increased Iba1 in the cornu ammonis 1 was observed in human tau pseudophosphorylated at 14 amino acid sites-infused rats at 1–3 but not 9 months post-infusion. Spatial learning deficiency in human tau pseudophosphorylated at 14 amino acid sites-infused rats at 1–3 months post-infusion was paralleled by decreased neuronal excitability, impaired NMDA receptor-dependent long-term potentiation and augmented L-type calcium channel-dependent long-term potentiation at the cornu ammonis 1 synapses. L-type calcium channel expression was elevated in the soma of the cornu ammonis 1 neurons in human tau pseudophosphorylated at 14 amino acid sites-infused rats. Chronic L-type calcium channel blockade with nimodipine injections for 6 weeks normalized neuronal excitability and synaptic plasticity and rescued spatial learning deficiency in human tau pseudophosphorylated at 14 amino acid sites-infused rats. The early onset of L-type calcium channel-mediated pretangle tau pathology and rectification by nimodipine in our model have significant implications for preclinical Alzheimer’s disease prevention and intervention.

## Teaser

Crossley *et al.* demonstrate experimentally, for the first time, that normalizing a calcium channel that is upregulated in a selective memory encoding brain structure in Alzheimer’s disease, could prevent spatial memory deficiency associated with pretangle tau, an abnormal protein implicated in preclinical Alzheimer’s disease.

## Graphical Abstract

**Graphical Abstract:**
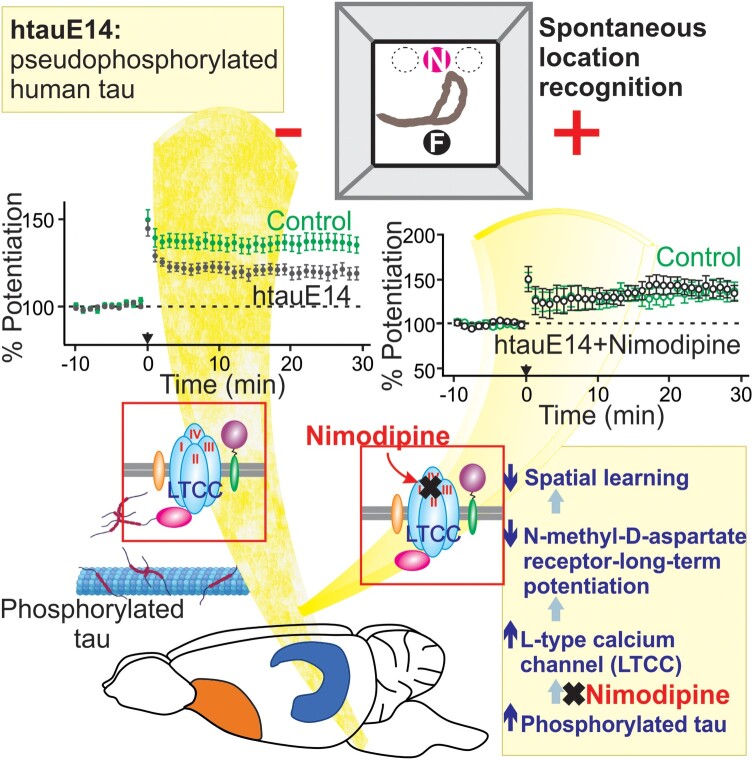

## Introduction

Alzheimer’s disease, the most common form of dementia in the world, still bears no cure. Therapeutic attempts using drugs such as cholinesterase inhibitors, and antibodies to remove aggregated proteins such as amyloid plaques, have yielded minimal results.^1,2^ There are compelling calls for interventions that potentially reverse deficits in Alzheimer’s disease subjects. As tau pathology is highly correlated with cognitive dysfunction in Alzheimer’s disease patients, targeting tau pathology appears to be a more promising approach.^2,3^

Tau pathology originates from soluble, abnormally persistently phosphorylated pretangle tau [precursor of neurofibrillary tangles (NFT)]. Pretangle tau, indexed by an AT8 antibody recognizing early phosphorylation at S202/T205, first appears in the locus coeruleus in human brains and subsequently spreads to other neuromodulatory nuclei and to the transentorhinal cortex, constituting Braak’s pretangle stages.^4^ Pretangle stages then transition to NFT stages, starting from the transentorhinal cortex and progressing to the hippocampus at Braak NFT Stage I/II. Clinical symptoms appear from Stage III/IV. The long prodromal period creates a permissive window for therapeutic intervention. Abnormally phosphorylated pretangle tau appears to be the earliest sign leading to Alzheimer’s disease.^4^ Recent research suggests that soluble pretangle tau, including oligomers, is more toxic than NFT,^3,5,6^ while animal studies suggest that NFT is not causal for cognitive decline or neuronal death and, in some cases, may even be neuroprotective.^6,7^

The long-standing calcium hypothesis of aging and Alzheimer’s disease proposes that sustained disruptions in calcium homeostasis contribute to altered synaptic plasticity, neural degeneration and impaired cognitive function.^8,9^ L-type calcium channels (LTCCs) are major contributors to excess intraneuronal calcium in aging and Alzheimer’s disease.^10,11^ Hence, LTCCs and their upstream and downstream factors can be therapeutic targets in Alzheimer’s disease. However, critical gaps in knowledge remain regarding how LTCCs are upregulated in Alzheimer’s disease and how LTCC hyperfunction relates to neuropathology.

In this study, we investigate the relationship between hyperphosphorylated pretangle tau and LTCC-mediated synaptic function by seeding pseudophosphorylated human 0N4R isoform of tau in the hippocampal cornu ammonis 1 (CA1), a structure that is selectively vulnerable to calcium dysregulation in Alzheimer’s disease patients and animal models.^12^ We found time-dependent changes of spatial learning, paralleled by alterations in neuronal and synaptic functions. Our findings shed light on cellular and synaptic mechanisms underlying the earliest pathology associated with Alzheimer’s disease.

## Materials and methods

### Ethics statement and subjects

**Figure 1:**
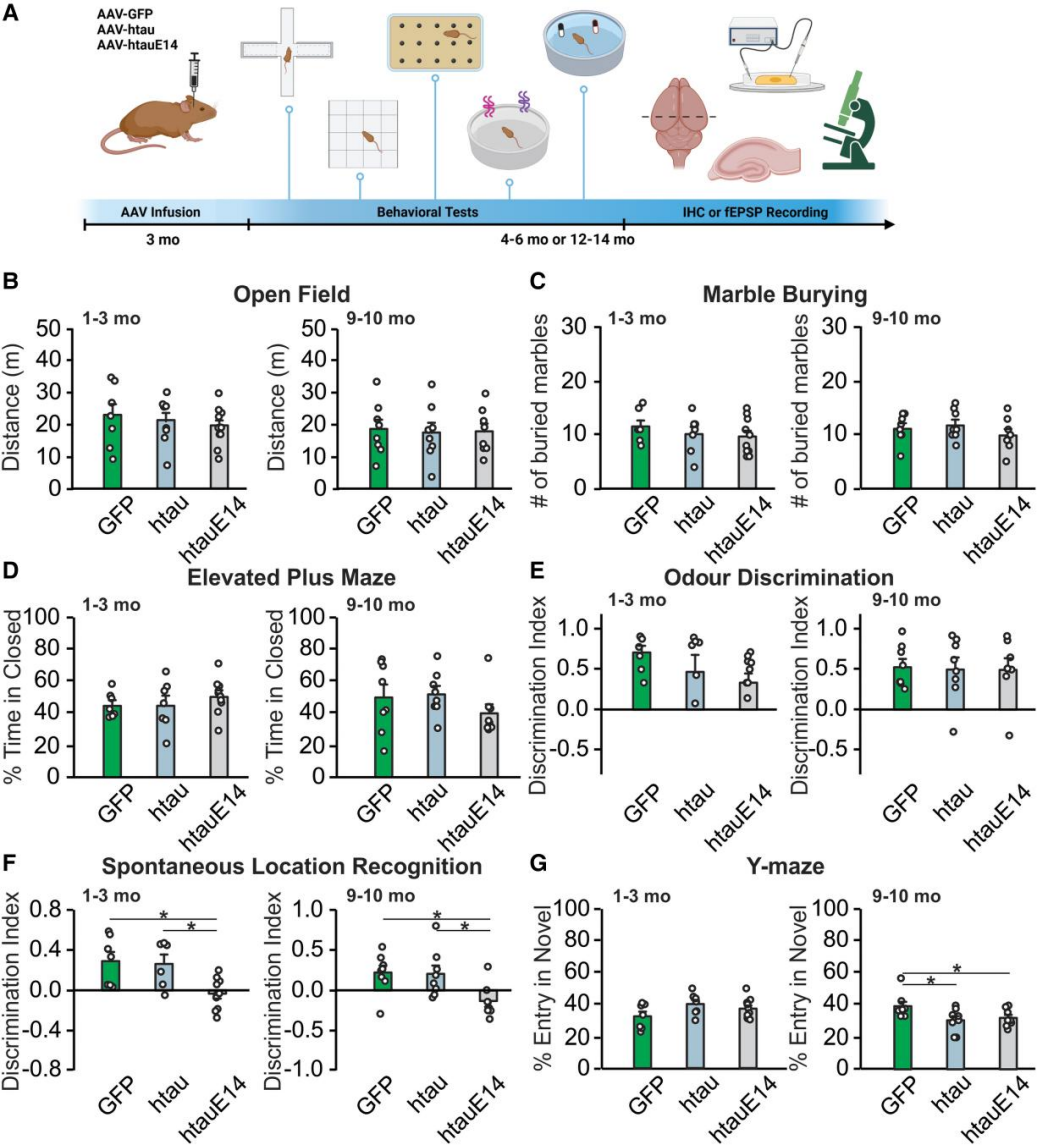
**CA1 htauE14 impairs spatial learning while htau-infused rats have milder deficiency.** (**A**) Schematics of experimental design (created with BioRender.com). (**B**) Distance travelled in an open field test at 1–3 months [*F*(2,22) = 0.383, *P* = 0.686; *N* = 7(3F + 4M)/ 8(4F + 4M)/10(3F+7M)] and 9–10 months post-infusion [*F*(2,21) = 0.040, *P* = 0.960; *N* = 8(3F + 5M)/8(3F + 5M)/8(5F + 3M)]. (**C**) Marbled buried in various groups at 1–3 months [*F*(2,21) = 0.669, *P* = 0.523; *N* = 7(3F + 4M)/7(4F + 3M)/10(3F + 7M)] and 9–10 months post-infusion [*F*(2,21) = 0.899, *P* = 0.422; *N* = 8(3F + 5M)/8(3F + 5M)/8(5F + 3M)]. (**D**) Percentage time spent in the closed arm of the elevated plus maze at 1–3 months [*F*(2,21) = 0.671, *P* = 0.522; *N* = 7(3F + 4M)/7(4F + 3M)/10(3F + 7M)] and 9–10 months post-infusion [*F*(2,21) = 1.103, *P* = 0.350; *N* = 8(3F + 5M)/8(3F + 5M)/8(5F + 3M)]. (**E**) Discrimination index [(sniffing time in the novel odour—sniffing time in the familiar odour)/sniffing time in the familiar odour] in a two similar odour discrimination test at 1–3 months [*F*(2,21) = 1.822, *P* = 0.186; *N* = 7(3F + 4M)/7(4F + 3M)/10(3F + 7M)] and 9–10 months post-infusion [*F*(2,21) = 0.021, *P* = 0.979; *N* = 8(3F + 5M)/8(3F + 5M)/8(5F + 3M)]. (**F**) Discrimination index [(time spent in the novel location—time spent in the familiar location)/time spent in the familiar location] in a spontaneous location recognition test at 1–3 months [*F*(2,20) = 6.605, *P* = 0.006; *N* = 7(3F + 4M)/6(3F + 3 M)/10(3F + 7M)] and 9–10 months post-infusion [*F*(2,21) = 5.065, *P* = 0.016; *N* = 8(3F + 5M)/8(3F + 5M)/8(5F + 3M)]. (**G**) Percentage entry numbers in a Y-maze at 1–3 months [*F*(2,21) = 2.242, *P* = 0.131; *N* = 7(3F + 4M)/7(4F + 3M)/ 10(3F + 7M)] and 9–10 months post-infusion [*F*(2,21) = 3.714, *P* = 0.042; *N* = 8(3F + 5 M)/8(3F + 5M)/8(5F + 3M)]. *P < 0.05; **P < 0.01. Sample numbers are presented as *N* = GFP/htau/htauE14. One-way ANOVAs were used for statistical analysis. Data points represent individual animals. F, female; M, male.

All experimental procedures were approved by the Institutional Animal Care Committee at Memorial University of Newfoundland and followed the Canadian Council’s guidelines on Animal Care. Wild-type Sprague–Dawley rats of both sexes were singly housed in a 12-h light/dark cycle and had *ad libitum* access to dry food pellets and water. Adeno-associated virus (AAV) infusions were conducted in 3-month-old rats. Behavioural experiments were carried out either 1–3 or 9–10 months post-infusion, followed by immunohistochemistry (IHC), histology or western blotting studies (Fig. 1A). A separate cohort was used for electrophysiological recordings 1–3 months post-infusion. Mixed sexes of rats were used in the experiments.

A separate cohort of animals received chronic subcutaneous nimodipine injections (5 mg/kg) 2–3 weeks post-AAV infusions, 5 days a week for 4 weeks prior to open field and spontaneous location recognition (SLR) behavioural tests. Injections continued concurrently with behavioural testing for 2 weeks. Injections and behavioural testing were conducted during the dark and light phases of the cycle, respectively, with an interval of 18 h to avoid acute drug effect. Behavioural tests were carried out by an experimenter blind to the injections. Following behavioural experiments, these animals were sacrificed for electrophysiological recording and Cav1.2 IHC. A total of 94 animals were used.

All procedures were designed to minimize pain and distress. We monitored rats closely following surgeries and used pain medication Meloxicam (0.5 mg/ml at 1 mg/kg) for surgery rats. Sufficient handling and habituation were conducted during behavioural experiments to minimize stress.

### Surgery

Animals were randomly assigned to hippocampal CA1 infusion surgeries at 2–3 months post-natal. One group was infused with an AAV containing a pseudophosphorylated human tau pseudophosphorylated at 14 amino acid sites (htauE14)^13^ under a CaMKII promoter (AAVdj-CaMKII-***htauE14***-EGFP). Another group was infused with AAV carrying human tau (AAVdj-CaMKII-***htau***-EGFP). AAVdj-CaMKII-EGFP was used for control. During surgeries, animals were anaesthetized using 3% isoflurane and mounted in a skull flat position using a stereotaxic apparatus. Bilateral holes were drilled in the skull for two CA1 infusion sites per hemisphere (Anterior-Posterior: −3.8 mm; and Medial-Lateral: ±1.6 mm and ±2.6 mm). An infusion cannula was lowered into the CA1 (Dorsal-Ventral: −2.2 mm). A 0.5-µl AAV was infused at each site for a total of 2 µl/animal, *via* a 10-µl Hamilton syringe attached to an infusion pump. Animals had a recovery period of at least 4 weeks prior to the start of behavioural study.

### Behavioural studies

#### Open field

To assess mobility, animals were placed in an open field maze measuring 60 × 60 × 50 cm^3^. They were given 10 min to freely explore the environment. The average speed and distance travelled were video recorded using ANYMaze software.

#### Marble burying

To assess compulsive behaviours, animals were placed in a clean cage containing 1 inch of bedding and 16 marbles arranged in a 4 × 4 grid. Following 30-min free exploration, animals were carefully removed from the cage to not disturb the marbles and the number of marbles buried at least 75% in bedding was counted.

#### Elevated plus maze

To assess anxiety-like behaviour, animals were placed in an elevated plus-shaped maze raised 50 cm above the ground. The arms each measure 50 × 10 cm^2^ and the two closed arms have a wall height of 38 cm surrounding the sides and end of the arm. During the 5-min trial, the amount of time the animals spent in the closed and open arms was recorded.

#### Odour discrimination

Discrimination of similar odours was tested using a perforated micro-centrifuge tube containing filter paper with 60 µl of odorant or mineral oil presented in 50-s trials separated by a 5-min intertrial interval. The first three trials used odourless mineral oil, the next three trials used Odour 1 (O1, 1-heptanol, 0.001%), and the last trial used an odour mixture that had a similar smell to O1 (O2, 1-heptanol and 1-octanol in a 1:1 ratio, 0.001%). The tests were videotaped and sniffing time within a 1-cm radius around the odour tube was measured offline. The discrimination index was the ratio of the sniffing time difference between the O2 and the third presentation of O1 to the total sniffing time (*t*_O2_ − *t*_O1–3_)/(*t*_O2_ + *t*_O1–3_).^13^

#### Spontaneous location recognition

During training, animals were placed in an open field chamber containing three identical cans at three locations and allowed to explore freely for 10 min. Twenty-four hours later, one can be remained in the same location (familiar location); the other two cans were removed and an identical can was placed in between the two locations (novel location). Animals were placed in the chamber for 10 min and the time they spent exploring the familiar and novel locations was recorded. The discrimination ratio was the difference between time spent at the novel and familiar objects over the total time spent on both objects (*t*_novel_ − *t*_familial_/*t*_total_).^13^

#### Y-Maze

Y-maze consisted of a black plexiglass Y-shaped chamber with three arms radiating from a central neutral zone. Each arm contains infrared sensors for automatic recording of the animal’s location in the maze. During training, one arm was blocked with a barrier, and animals were given 15 min to freely explore two open arms. Four hours later, the animals were put in the Y-maze for 15 min to freely explore all three arms. The time and number of entries into each arm were recorded.

### Electrophysiological recordings

#### Slice preparation

Animals were anaesthetized with isoflurane and decapitated and brains were extracted. Coronal slices of 400 µm containing dorsal hippocampus were cut using a vibratome (Leica VT-1200S) in an ice-cold cutting solution (in mM, 92 NaCl, 2.5 KCl, 1.2 NaH2PO4, 30 NaHCO3, 20 HEPES, 25 glucose, 5 sodium ascorbate, 2 thiourea, 3 sodium pyruvate, 10 MgSO4 and 0.5 CaCl2) bubbled with carbogen gas (95% O_2_/5% CO_2_). Slices were recovered at 34° for 30 min in an interface incubation chamber, with a filter membrane placed on top of a beaker containing aCSF (in mM: 124 NaCl, 2.5 KCl, 1.2 NaH2PO4, 24 NaHCO3, 5 HEPES, 12.5 glucose, 2 MgSO4 and 2 CaCl2) bubbled with carbogen gas. Slices were then recovered for a minimum of 2 h at room temperature before recording.

#### fEPSP recordings

Slices were placed in an open chamber containing a continuous flow of carbogenated aCSF containing (in mM): 119 NaCl, 5 KCl, 4 CaCl_2_, 4 MgSO_4_, 1 NaH_2_PO_4_, 26.2 NaHCO_3_, 22 glucose and 0.1 picrotoxin maintained at 28–30°C. The high divalent concentrations (4 mM Ca^2+^ and 4 mM Mg^2+^) were used to suppress epileptiform activity in the presence of the GABA_A_ receptor antagonist picrotoxin.

A concentric stimulation electrode was placed in the Schaffer collateral of the CA1 and a recording electrode filled with aCSF was placed in the same layer. An input–output curve was generated by recording field excitatory postsynaptic potentials (fEPSPs) with incremental stimulation intensities from 0 to 120 μA in intervals of 20 μA. Baseline stimulations were chosen at 50–70% of the maximum fEPSP slope. Paired-pulse ratio was recorded with two stimulations at 50-ms intervals. For long-term potentiation (LTP) recording, following a stable baseline (stimulations that generated ∼50% maximum fEPSP), a theta-burst stimulation (TBS) protocol was used (10 bursts of stimulation at 5 Hz, each burst containing five pulses at 100 Hz) to elicit N-methyl-D-aspartate receptor (NMDAR)-dependent LTP. To elicit compound LTP (LTCC- and NMDAR-dependent), a 200-Hz protocol was used (10 bursts of 200 Hz, 200 ms every 5 s).^14^ D-APV (50 μM) was used to isolate the LTCC-LTP. Two to four recordings were obtained from each animal. Data were acquired using MultiClamp 700B and filtered at 2 kHz and digitized at 10 kHz using pClamp 10.5 software. Offline analysis of fEPSP slopes was conducted using ClampFit 10.5.

### IHC, histology and western blotting

IHC of LTCC subunit Cav1.2, NMDAR subunits GluN1, PSD-95, HT7, AT8, Iba1 and GFP, Nissl histology, and thioflavin-S staining followed established protocols.^13,15^ T22, ptau S262/T263, ptau S356 and Tau5 were used for detecting tau oligomerization and phosphorylation with WB.

#### IHC and histology

Animals were anaesthetized with pentobarbital (80 mg/kg) and perfused transcardially with ice-cold 4% paraformaldehyde. Brains were collected and stored in 4% paraformaldehyde. Coronal sections of 50 μm were collected using a Leica VT 1000S vibratome and were stored at 4°C in a PVP solution.

For fluorescence antibody staining, free-floating sections were washed 2 × 10 min in Tris buffer, followed by 10-min washes in Tris A (0.1% Triton-X-100 in Tris buffer) and Tris B (0.1% Triton-X and 0.005% bovine serum albumin [BSA] in Tris buffer), and then incubated overnight in the cold room in 5% bovine serum albumin in Tris B with GluN1 (1:500; Cell Signalling 5704S) together with PSD-95 (1:1000; Invitrogen MA1-046). Sections were washed for 10 min each in Tris A and Tris B and then incubated for 1 h in 5% bovine serum albumin in Tris B with Alexa Fluor 647 goat anti-rabbit immunoglobulin G (1:1000; Invitrogen A21244) and Alexa Fluor 555 goat anti-mouse immunoglobulin G (1:1000; Invitrogen A21424), followed by a 10-min wash in Tris buffer. Cav 1.2 staining and co-labelling with PSD95 were conducted in separate steps.^15^ After secondary antibody staining and washing, sections were floated onto slides and treated for 30 s with TrueBlack Lipofuscin Autofluorescence Quencher (1×, prepared in 70% ethanol, Biotium, 23007) and then washed 3 × 5 min in phosphate-buffered saline before being coverslipped using Fluoroshield Mounting Medium with 4′,6-diamidino-2-phenylindole (Abcam AB104139).

For 3,3ʹ-diaminobenzidine staining, sections were washed 3 × 5 min in Tris buffer to remove PVP. Sections were then washed in a 1% hydrogen peroxide solution in Tris buffer for 30 min. Sections underwent a series of washes 1 × 5 min in Tris buffer, 1 × 10 min in Tris A and 1 × 10 min in Tris B. Sections were then washed in a 10% normal goat serum in Tris B solution for 1 h after which they were washed 1 × 10 min in Tris A and 1 × 10 min in Tris B. Sections were placed in primary antibody [GFP (1:2000; Invitrogen A11122), Iba1 (1:2000; Wako 019-19741), HT7 (1:1000; Invitrogen, MN1000) or AT8 (1:1000; Invitrogen, MN1020)] in Tris B and incubated on a shaker for 48 h at 4°C. Following the incubation, sections were washed in Tris A and Tris B for 1 × 10 min and then placed in biotinylated secondary antibody at a concentration of 1:1000 in Tris B for 45 min. Sections were washed 1 × 10 min in Tris A and 1 × 10 min in Tris D (0.1% Triton-X and 0.005% bovine serum albumin in 0.5 M Tris buffer). This was followed by a 90-min incubation in 1% A + B (avidin–biotin complex) in Tris D. Sections were washed 3 × 5 min in Tris buffer before being transferred to a 3,3ʹ-diaminobenzidine solution (50-ml Tris buffer, 50-ml water, 50-mg 3,3ʹ-diaminobenzidine and 30-µl hydrogen peroxide) for up to 30 min. Sections were washed one last time in Tris buffer and were mounted on chrome–gelatin-coated slides. Slides were left to dry overnight and were dehydrated the following day using increasing ethanol concentrations (70%, 95% and 100%) for 2 min each. They were then cleared in xylene for 2 min after which they were coverslipped with Permount.

For Nissl staining, sections were mounted onto slides that were left to dry overnight. Slides were placed in decreasing concentrations of ethanol (100%, 95% and 70%) for 2 min each before being dipped in distilled water (dH_2_O) to rehydrate the tissue. Slides were then submerged in 1% cresyl violet for up to 15 min. After rinsing with dH_2_O, slides were dehydrated using increasing ethanol concentrations (70%, 95% and 100%) for 2 min each. Slides were then cleared in xylene for 2 min after which they were coverslipped with Permount.

For thioflavin-S staining, slides were first heated at 90°C for 10 min to quench the GFP signal. Slides were defatted in xylene for 5 min, followed by rehydration in serial dilutions of ethanol (100%, 95%, 70% and 50%; 3 min each) and water (2 × 3 min). Then, slides were incubated for 8 min in a filtered 1% aqueous solution of thioflavin-S (Sigma) in the dark at room temperature. Washes with ethanol (70%, 2 × 3 min; 95%, 3 min) and water (3 exchanges) followed before mounting with aqueous mounting media (Sigma).

#### Imaging and analysis

Using a BX53 brightfield microscope (Olympus), images were taken of all 3,3ʹ-diaminobenzidine and Nissl-stained slides at a magnification of 20×. Positively stained cells were manually counted within ImageJ software by a blind experimenter. Thioflavin-S images were acquired by an EVOS M5000 imaging system (Thermo Fisher Scientific).

Using a confocal microscope (Zeiss LSM 900 with Airyscan 2), images were taken of the fluorescent-stained slides. Images of the dorsal hippocampal CA1 pyramidal cell layer were taken at a magnification of 20× with 8× zoom. Using ImageJ, within the CA1 somatic cell layer, regions of interest were chosen from GFP^+^ cells, and for each region of interest, integrated fluorescence intensity was measured. Ten GFP^+^ cells cross the CA1 layer were randomly chosen, and the fluorescence intensity was averaged for each slice. For dendritic puncta measurement, MATLAB was used to count puncta and percentage of co-localization with PSD95. Images were converted to 8 bits and were loaded into the MATLAB environment using the imread function. Median filtering was applied to both channels to reduce noise and enhance the quality of the images. Grey thresholding was used to create binary masks for each channel. Binary masks were labelled, and the number of puncta in each channel was determined. Binary images from both channels were then overlaid and a new image was generated with co-localized puncta. The percentage of co-localized puncta (synaptic) over the total number of the PSD95 puncta was calculated.

#### Western blotting

Following decapitation, bilateral hippocampal tissues extracted from rats were weighed, flash frozen and stored at −80°C. Frozen brain samples were homogenized using a mortar on ice with lysis buffer (Thermo Scientific, Pierce RIPA Buffer, 89901; 1 ml of lysis for every 40-mg sample weight). Ethylenediaminetetraacetic acid-free 1× phosphatase and protease inhibitors (Thermo Scientific, 87786 and 78420) were added to the lysis buffer during tissue homogenization to prevent protein degradation and centrifuged at 13,500 rpm for 30 min at a 4°C temperature. The supernatant was aliquoted into new centrifuge tubes and stored at −80°C.

Protein estimation was obtained using the standard Pierce BSA Protein Assay Kit (Thermo Scientific 23225), and absorbance was read at the 562-nm wavelength. Protein lysates were thawed on ice and mixed in a 3:1 ratio with 4× Laemmli buffer (containing 0.5 M Tris-HCl, 50% glycerol, 0.25% Sodium Dodecyl Sulfate, 0.25% bromophenol blue, beta-mercaptoethanol and 1× cocktail protease and phosphatase inhibitors). Samples were vortexed briefly and heated at 95°C for 5 min. For the detection of T22, samples were prepared without beta-mercaptoethanol and heating.

Twenty micrograms of protein samples were loaded and separated on a 10% (7.5% for T22) sodium dodecyl sulphate-polyacrylamide gel electrophoresis and transferred to Immobilon-P Transfer polyvinylidene difluoride (PVDF) membranes (Merck Millipore, IPVH00010). PVDF membranes were pre-wet in 100% methanol for 10 s to allow membrane activation and then soaked in distilled water for ∼2 min, followed by equilibration in 1× transfer buffer. The transfer apparatus was set up in the cold room (4°C), and the transfer was done for 60 min (90 min for Tau22). The blots were briefly rinsed with 1× low salt TBS-T (containing 1.5 M NaCl, 1 M Tris Base and 0.1% Tween 20) and blocked with 5% non-fat dry skim milk diluted in low salt TBS-T buffer for 1 h at room temperature. Primary antibodies included Tau5 (1:3000, Millipore 577801), T22 (1:2000; Millipore ABN454), pS262/T263 (1:2000; Abcam ab92627), pS356 (1:5000; Abcam ab75603) and β-actin (1:2000; Invitrogen AM4302) were diluted in the blocking buffer containing 0.1% Tween 20 and incubated on a rocker at room temperature for 2 h. Blots were rinsed in high salt TBS-T (containing 5 M NaCl, 1 M Tris Base and 0.1% Tween 20) 2 × 5 min and a 1 × 5 min rinse in low salt TBST to remove non-specific binding. Secondary antibody incubation occurred at room temperature for 1 h with either horse-radish peroxidase-labelled anti-rabbit immunoglobulin G (H + L, 31460) or horse-radish peroxidase-labelled anti-mouse immunoglobulin G (31430) diluted in low salt TBST at a 1:4000 concentration. Blots were rinsed in high salt TBST (2 × 5 min) and a 1 × 5 min rinse in low salt TBST. The protein bands were visualized using a chemiluminescent substrate (Thermo Fisher Supersignal West PICO, 34577) on a digital image scanner (ImageQuant LAS 4000) and quantified using ImageJ software. Samples were normalized to the β-actin protein.

### Statistical analysis

All statistical analyses were completed using OriginPro 2022b. One-way or two-way ANOVAs with *post hoc* Tukey tests were used to compare more than two groups. Student *t*-tests (two-tailed) were used for two group comparisons. Pearson correlation coefficient was used to measure the correlation. Equal variance was assumed. Data are presented as mean ± SEM. Sample sizes were chosen based on previously published work.^13,16^

## Results

### Hyperphosphorylated human tau infusion in the CA1 impairs spatial learning

Three groups of rats were included for behavioural tests: an htauE14-infused group (AAVdj-CaMKII-***htauE14***-EGFP), an htau-only group (AAVdj-CaMKII-***htau***-EGFP) and an EGFP control group (AAVdj-CaMKII-EGFP). We conducted a battery of behavioural tasks at two time points (1–3 or 9–10 months) following infusions of AAVs in the CA1 (Fig. 1A). At both time points, no behavioural differences were observed amongst groups in the distance travelled in the open field (Fig. 1B), marbles buried (Fig. 1C), time spent in the closed arm of the elevated plus-shaped maze (Fig. 1D) and odour discrimination index (Fig. 1E). However, htauE14-infused rats showed impaired SLR (significantly lower discrimination index) compared with the htau and GFP groups at both 1–3 and 9–10 months post-infusion (Fig. 1F). Both htau and htauE14 groups were impaired in the Y-maze test (fewer entry to the novel arm compared with control) at 9–10 months, but not at 1–3 months post-infusion. These results suggest that tau hyperphosphorylation-induced neural toxicity in CA1 and behavioural impairment occur earlier and are more severe than the deficiency induced by human tau expression in rats.

### CA1 htauE14 infusion results in tau hyperphosphorylation but no tangle formation

**Figure 2:**
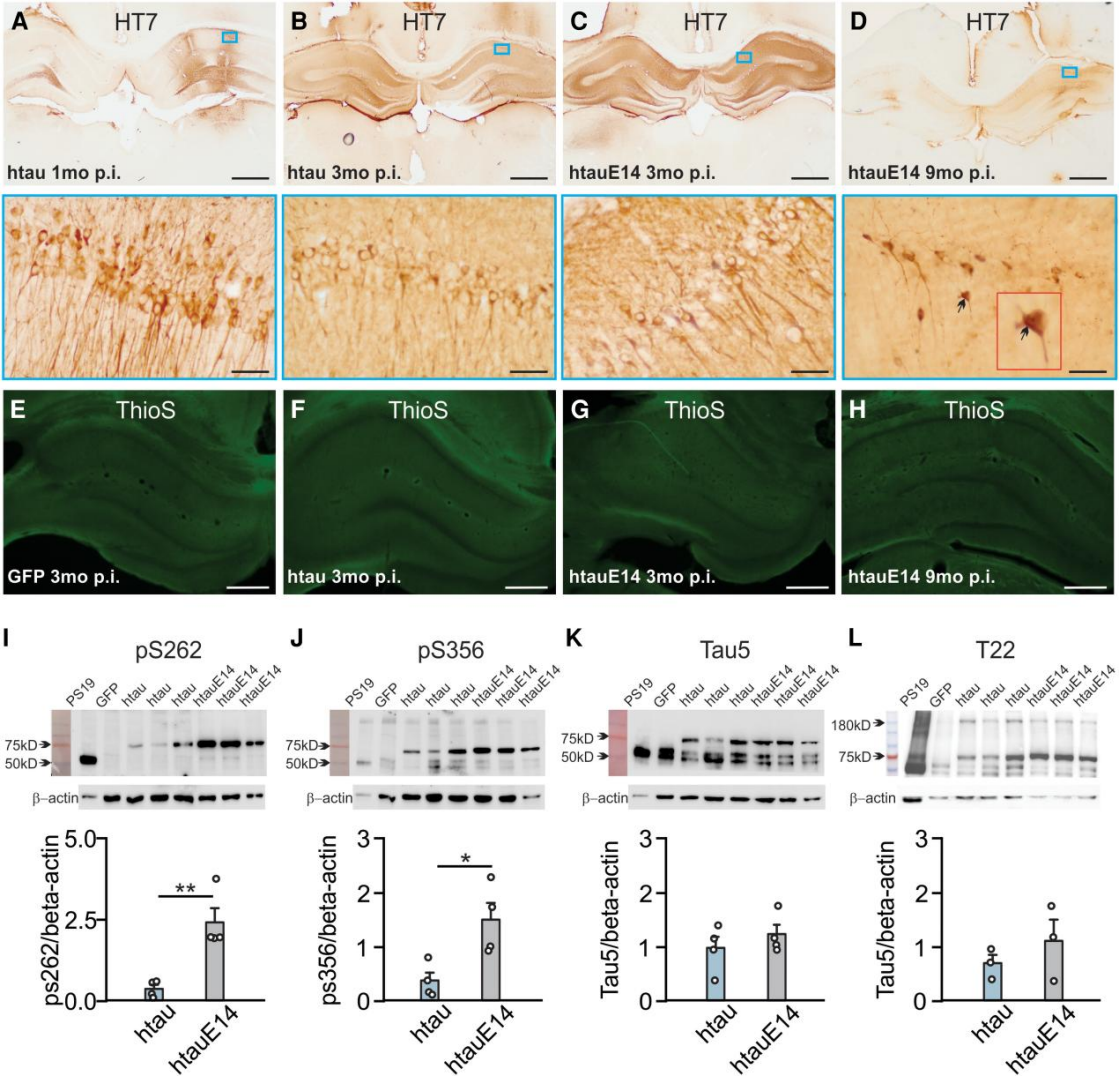
**Increased phosphorylation induced by htauE14 infusion at 3 months post-infusion.** (**A–D**) Human tau HT7 expression in CA1 neurons in htau-infused rats 1 month p.i. (**A**) and 3 months p.i. (**B**) and htauE14-infused rats at 3 months p.i. (**C**) and 9 months p.i. (**D**). Lower panels are zoom-in images of the small squares in the upper panels. Scale bars: 500 µm (zoom in 50 µm). Arrows in **D** indicate a cell with nucleolar tau expression. (**E–H**) Thioflavin-S (ThioS) staining in the hippocampi of different groups at various time points: (**E**) a GFP-infused brain 3 months p.i.; (**F**) an htau-infused brain 3 months p.i.; (**G**) an htau-E14-infused brain 3 months p.i.; and (**H**) an htau-E14-infused brain 9 months p.i. Note negative staining at all time points. Scale bars: 500 µm. (**I–L**) pS262 [*t* = 4.361, *P* < 0.01; *N* = 4(2F + 2M)], pS356 [*t* = 3.137, *P* = 0.020; *N* = 4), Tau5 (*t* = 0.899, *P* = 0.403; *N* = 4(2F + 2M)] and T22 [*t* = 0.948, *P* = 0.396; *N* = 3(1F + 2M)] expressions in the hippocampal tissue of htau and htauE14 brains. pS262, pS356 and Tau 5 were measured at 75-kD bands. T22 was measured at 180-kD bands. Upper panels: WB example bands. Uncropped blots of the target proteins were used. Lower panels: tau expressions normalized to β-actin. *P < 0.05; **P < 0.01. Two-tailed *t*-tests were used for statistical analysis. Data points represent individual animals. See Supplementary Material Fig. 5 for uncropped western blots. F, female; M, male; p.i., post-infusion.

The spread patterns of htauE14 in the hippocampus indexed by GFP staining were similar in 1–3 and 9–10 months brains (Supplementary Fig. 1). GFP expression rates in the CA1 were 17.71 ± 1.01% in 1–3 months and 31.20 ± 5.83% in 9–10 months post-infusion. Individual transduction rate was not correlated with spatial learning performance (Supplementary Fig. 2), suggesting htauE14 expression as low as 10–15% in CA1 is sufficient to impair spatial learning. HT7 (Fig. 2A–D) and AT8 (indexing pretangle tau phosphorylated at S202/T205; Supplementary Fig. 3) staining showed pathological patterns in 9 months post-infusion brains compared with 3 months, with nucleolar expression and atrophic processes,^17^ parallel to more severe spatial learning deficiency observed at 9 months post-infusions. No neurofibrillary tangle or amyloid plaque formation was observed (Fig. 2E–H; positive control see Supplementary Fig. 4). However, Tau phosphorylations at S262 and S365 (two microtubule binding sites) were significantly higher in htauE14 brains than in htau brains at 3 months post-infusion, although the T22 oligomer levels were similar (Fig. 2I–L; Supplementary Fig. 5). These results suggest that pretangle hyperphosphorylated tau, but not tau tangles in the hippocampus, is associated with spatial learning deficiency.

### No cell loss but transient enhanced Iba1 activation is associated with htauE14

**Figure 3:**
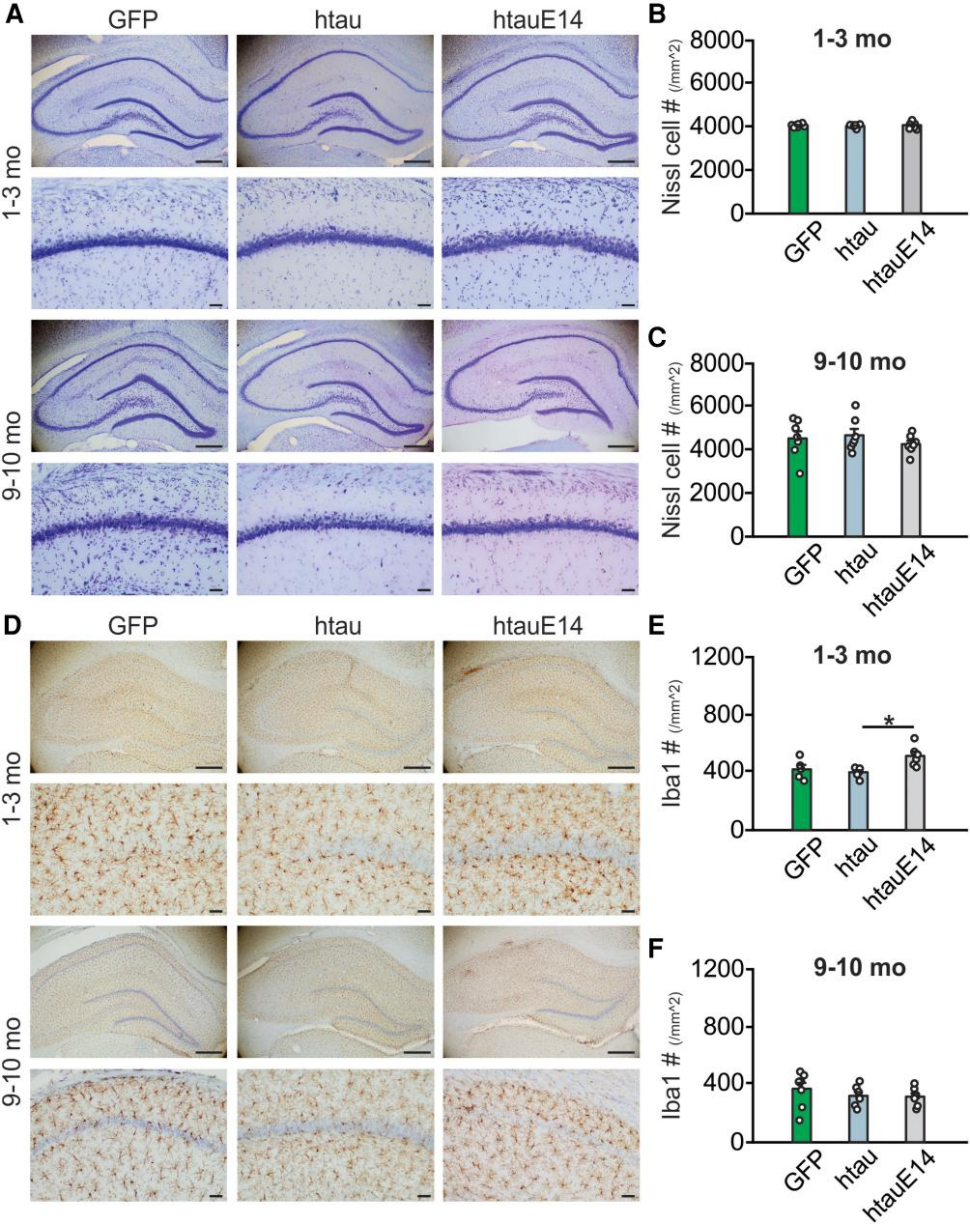
**Early increase in Iba1 expression but no cell loss in htauE14-infused rats.** (**A**) Example images of Nissl staining of GFP, htau and htauE14 hippocampus at 1–3 months post-infusion (upper two panels) and 9–10 months post-infusion (low two panels). Zoom-in CA1 images are shown below the full hippocampal images. (**B**) Nissl cell counts at 1–3 months post-infusion [*F*(2,20) = 0.239, *P* = 0.799; *N* = 7(3F/4M)/7(4F/3M)/9(2F/7M)]. (**C**) Nissl cell counts at 9–10 months post-infusion [*F*(2,18) = 0.578, *P* = 0.571; *N* = 7(3F/4M)/7(3F/4M)/7(4F/3M)]. (**D**) Example images of Iba1 staining of GFP, htau and htauE14 hippocampus at 1–3 months post-infusion (upper two panels) and 9–10 months post-infusion (low two panels). Zoom-in CA1 images are shown below the full hippocampal images. (**E**) Iba1 cell counts at 1–3 months post-infusion [*F*(2,14) = 5.836, *P* = 0.014; *N* = 5(1F/4M)/6(3F/3M)/6(1F/5M)]. (**F**) Iba1 cell counts at 9–10 months post-infusion [*F*(2,19) = 0.906, *P* = 0.421; *N* = 8(3F/5M)/7(3F/4M)/7(4F/3M)]. Scale bars: 500 and 50 µm (zoom-in images). *P < 0.05. Sample numbers are presented as *N* = GFP/htau/htauE14. One-way ANOVAs were used for statistical analysis. Data points represent individual animals. F, female; M, male.

Despite spatial learning deficiency observed as early as 1–3 months post-infusion, and the appearance of pathological tau patterns at 9 months post-infusion of htauE14, there was no significant cell loss based on Nissl cell counts (Fig. 3A–C). However, increased Iba1 expression in the CA1 layer was observed with the htauE14 infusion at 1–3 months post-infusion, but not at 9–10 months post-infusion (Fig. 3D–F; Supplementary Fig. 6), suggesting an early activation of microglia may play a protective role.

### CA1 htauE14 results in impaired NMDAR synaptic plasticity and LTCC hyperfunction at 1–3 months post-infusion

**Figure 4:**
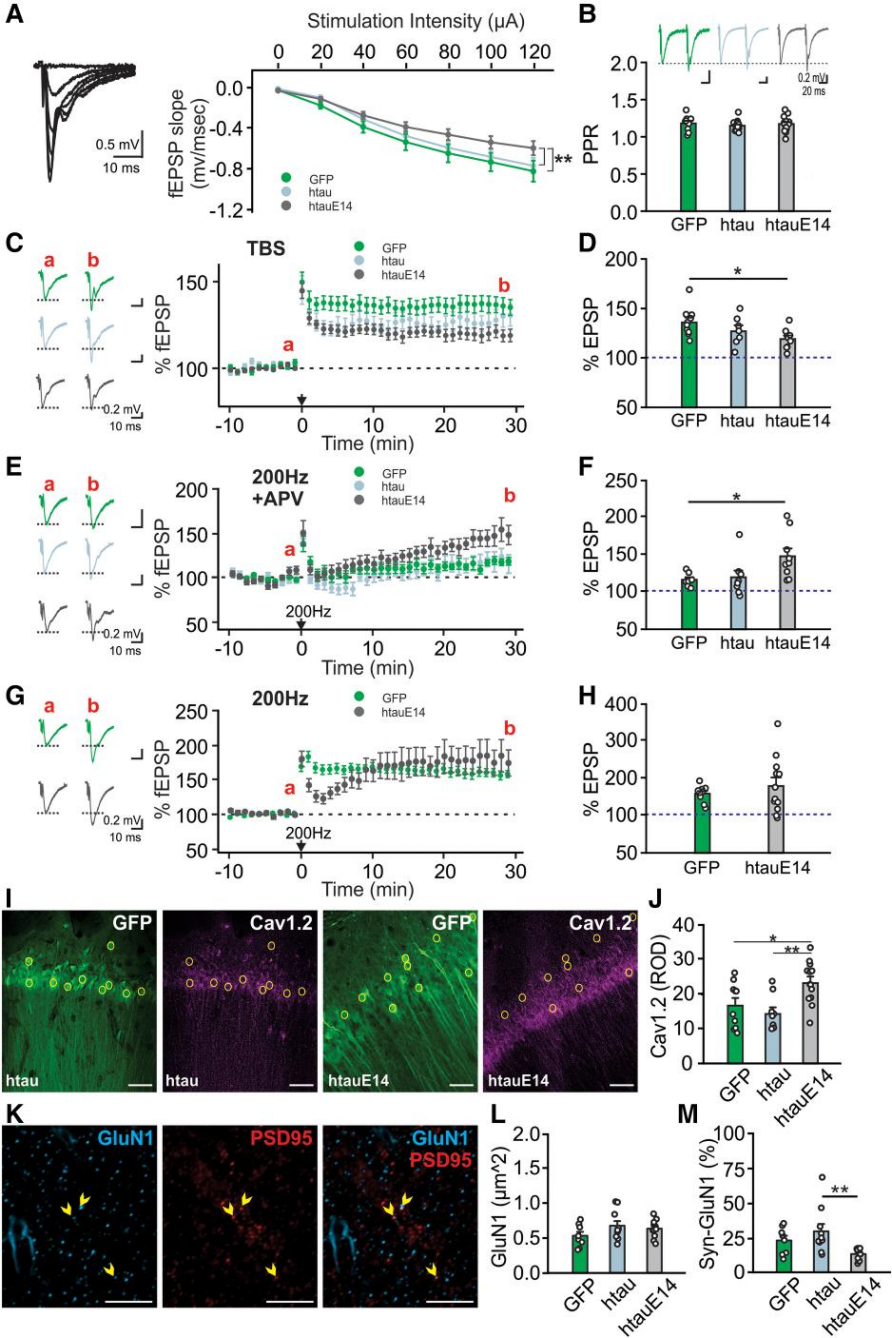
**CA1 htauE14 transduction for 1–3 months impairs NMDAR-dependent LTP and augments LTCC function.** (**A**) Input–output relationship of fEPSP slope versus stimulation intensity showed impaired excitability in htauE14 CA1 [*F*(2,20) = 10.377, *P* < 0.01; *N*_slice_ = 7/8/9 from *N*_animal_ = 5(4F/1M)/3(1F/2M)/4(3F/1M)]. (**B**) PPRs were not different amongst groups [*F*(2,36) = 0.408, *P* = 0.668; *N*_slice_ = 9/14/16 from *N*_animal_ = 5(4F/1M)/5(2F/3M)/7(4F/3M)]. (**C**, **D**) TBS-induced LTP was impaired in htauE14 group [*F*(2,23) = 3.941, *P* = 0.034; *N*_slice_ = 10/7/9 from *N*_animal_ = 5(3F/2M)/3(1F/2M)/4(3F/1M)]. (**E**, **F**) LTCC-dependent LTP was enhanced by htauE14 [*F*(2,21) = 4.083, *P* = 0.032; *N*_slice_ = 7/8/9 from *N*_animal_ = 3(3F)/3(2F/1M)/3(2F/1M)]. APV, (2R)-amino-5-phosphonovaleric acid. (**G**, **H**) Compound LTP induced by 200-Hz induction had similar amplitudes between htauE14 and GFP animals [*t* = 0.924, *P* = 0.365; *N*_slice_ = 12/12 from *N*_animal_ = 5(4F/1M)/7(3F/4M)]. (**I**) Example images of Cav1.2 and GFP in the CA1 of an htau brain (left two panels) and an htauE14 brain (right two panels). Circles indicate selected GFP^+^ cells for analysis. (**J**) Relative optical density of various groups [*F*(2,29) = 6.988, *P* = 0.003; *N*_slice_ = 10/9/13 from *N*_animal_ = 4(1F/3M)/3(/3M)/4(1F/3M)]. Scale bars: 50 µm. (**K**) GluN1 and post-synaptic density protein (PSD95) labelling in the CA1 dendritic layer. Arrows indicate co-labelled puncta. Scale bars: 5 µm. (**L)** Density of GluN1 puncta was not different amongst groups [*F*(2,26) = 1.657, *P* = 0.210; *N*_slice_ = 8/10/11 from *N*_animal_ = 5(3F/2M)/6(5F/1M)/2F/5M)]. (**M**) Synaptic GluN1 (Syn-GluN1) puncta (% of PSD^+^ puncta) was lower in htauE14 group [*F*(2,26) = 5.528, *P* = 0.010; *N*_slice_ = 8/10/11 from *N*_animal_ = 5(3F/2M)/6(5F/1M)/2F/5M)]. *P < 0.05; **P < 0.01. Sample numbers are presented as *N* = GFP/htau/htauE14 (*N* = GFP/htauE14 in **H**). One-way ANOVAs or two-tailed *t*-tests (two-group comparison in **H**) were used for statistical analysis. Data points are mean ± SEM. Data points represent individual slices and animal numbers are indicated as above. F, female; M, male; PPR, paired-pulse ratio.

We next examined neuronal excitability and synaptic plasticity in the CA1 of the dorsal hippocampus using *ex vivo* fEPSP recordings in separate cohorts. CA1 neuronal excitability was reduced in htauE14 animals compared with the htau and EGFP controls (Fig. 4A; Supplementary Fig. 7), while presynaptic release indexed by paired-pulse ratios was comparable (Fig. 4B). TBS-induced LTP, which was NMDAR dependent (Supplementary Fig. 8), was impaired in the htauE14 group (Fig. 4C and D). However, an LTCC-dependent LTP (200-Hz induction in the presence of APV) was enhanced at CA1 synapses in htauE14 animals (Fig. 4E and F). In the absence of APV, 200-Hz induction resulted in a compound LTP including both NMDAR- and LTCC-dependent components (Supplementary Fig. 8)^14^ and was similar in amplitudes at the last 5 min between the htauE14 and GFP groups (Fig. 4G and H). However, the LTP in htauE14 animals exhibited a unique time course form, with an initial rebound response following the immediate potentiation, and a slow-growing LTP that reached a plateau at the end of the recording. The waveform resembles that recorded in the presence of APV, likely reflecting a deficiency in the NMDAR component of the LTP.

We have previously shown an aging-associated increase in Cav1.2 expression in the somatic membrane of CA1 neurons and parallel decrease in CA1 neuronal excitability.^15^ Here, we measured Cav1.2 expression in CA1 neurons and observed increased expression in the somatic membrane in htauE14-expressing neurons compared with htau or GFP animals (Fig. 4I and J). The synaptic (co-localizing with PSD-95) and extra-synaptic expression levels were comparable in different groups (Supplementary Fig. 9). We then measured NMDAR GluN1 subunit expression in the CA1 layer (Fig. 4K–M, Supplementary Fig. 10). Neither dendritic (Fig. 4L) nor somatic GluN1 expression (Supplementary Fig. 10) was different amongst groups. However, there was a reduction of synaptic GluN1 expression as measured by its co-localization with PSD95 (Fig. 4M), suggesting a shift of GluN1 receptors from synaptic to extra-synaptic sites. PSD95 density was similar amongst groups (Supplementary Fig. 11).

### Chronic blockade of LTCCs in the CA1 rescues spatial learning deficiency, restores neuronal excitability and normalizes synaptic plasticity

**Figure 5:**
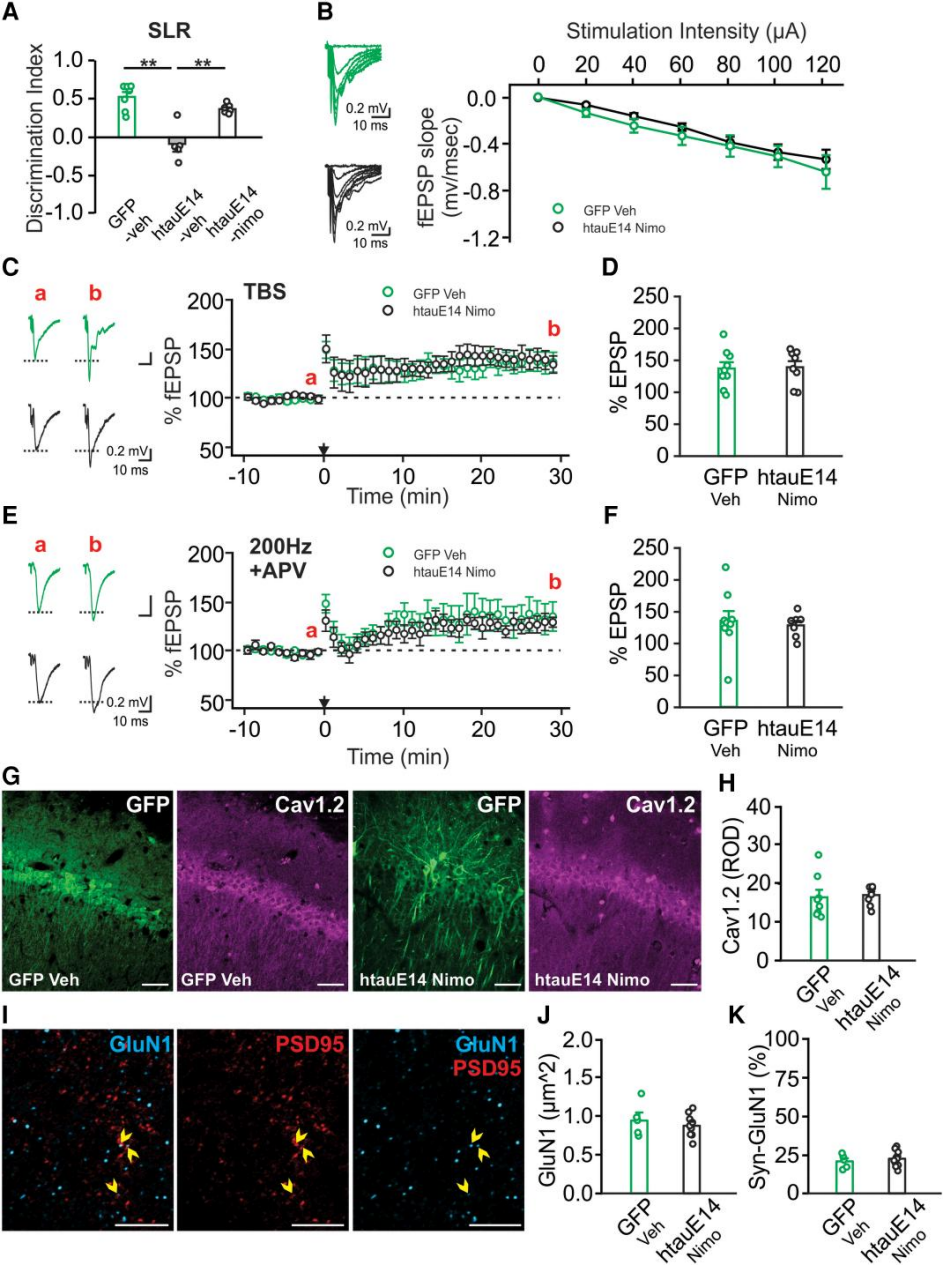
**Chronic nimodipine injection rescues spatial learning deficiency and normalizes synaptic plasticity.** Nimodipine was systemically injected for 6 weeks before and through the behavioural tasks. (**A**) Discrimination index of a SLR test [*F*(2,16) = 22.724, *P* < 0.01; *N* = 7(1F/6M)/5(2F/3M)/7(4F/3M)]. (**B**) Input–output relationship of fEPSP slope versus stimulation intensity was similar in htauE14 rats injected with nimodipine (htauE14 Nimo) and GFP vehicle (GFP Veh) control [*F*(1,13) = 2.904, *P* = 0.09; *N*_slice_ = 11/11 from *N*_animal_ = 3(3M)/3(3F)]. (**C**, **D**) TBS-induced LTP was normal in htauE14 Nimo rats [*t* = 0.141, *P* = 0.890; *N*_slice_ = 9/8 from *N*_animal_ = 3(3M)/3(3F)). (**E**, **F**) LTCC-dependent LTP was comparable in htauE14 Nimo group compared with GFP Veh control [*t* = 0.373, *P* = 0.714; *N*_slice_ = 9/7 from *N*_animal_ = 3(1F/2M)/3(3F)]. (**G**) Example images of Cav1.2 staining in a GFP Veh brain (left two panels) and an htauE14 Nimo brain (right two panels). (**H**) Comparison of Cav1.2 somatic expression in the two indicated groups [*t* = 0.275, *P* = 0.787; *N*_slice_ = 8/8 from *N*_animal_ = 2(2M)/2(1F/1M)]. Scale bars: 50 µm. (**I**) GluN1 and PSD95 labelling in the CA1 dendritic layer. Arrow heads indicate co-labelled puncta. Scale bars: 5 µm. (**J)** Density of GluN1 puncta was not different between the GFP Veh and htauE14 Nimo groups [*t* = 0.734, *P* = 0.476; *N*_slice_ = 5/10 from *N*_animal_ = 2(2M)/4(1F/3M)]. (**K**) Synaptic GluN1 puncta (% of PSD^+^ puncta) was similar in the two groups [*t* = 0.625, *P* = 0.542; *N*_slice_ = 5/10 from *N*_animal_ = 2(2M)/4(1F/3M)). * *P* < 0.05; ** *P* < 0.01. Sample numbers are presented as *N* = GFP veh/htauE14 veh/htauE14 nimo (**A**) or *N* = GFP veh//htauE14 nimo (**B–K**). Two-tailed *t*-tests were used for statistical analysis. Data points represent individual animals in **A** and individual slices in **D**, **F**, **H**, **J** and **K**. F, female; M, male.

Impaired NMDAR-dependent plasticity in the CA1 could lead to spatial learning deficiency such as what we observed in the SLR task. Indeed, SLR was dependent on NMDAR activation in the CA1 and was impaired when APV was infused into the CA1 prior to the training (Supplementary Fig. 12). In aging population, LTCC blockers have shown beneficial effects in reversing neuronal dysfunction and cognitive impairment in both humans and animal models.^18-20^ Here, we injected LTCC antagonist nimodipine within 1 month post-AAV infusion, for 4 weeks prior to, and throughout the behavioural tasks. Chronic nimodipine injection did not affect the movement of the animals (Supplementary Fig. 13) but reversed the SLR deficiency observed in vehicle-injected htauE14 rats (Fig. 5A). Neuronal excitability in the CA1 of nimodipine-injected htauE14 rats was comparable with the vehicle-injected GFP rats (Fig. 5B). Both NMDAR-dependent LTP (Fig. 5C and D) and LTCC-dependent LTP (Fig. 5E and F) showed comparable increase in fEPSP slopes between the two groups. Chronic nimodipine injection also prevented Cav1.2 somatic overexpression (Fig. 5G and H). Interestingly, nimodipine treatment also normalized GluN1 distribution such that there was no difference of synaptic GluN1 expression between the GFP and htauE14 groups (Fig. 5I–K).

## Discussion

Our results revealed that hyperphosphorylated tau in CA1 neurons led to impaired NMDAR-dependent synaptic plasticity and spatial learning following a short period of htauE14 seeding (1–3 months), which was mediated by upregulation of LTCCs and concomitant shift of GluN1 subunit from synaptic and extra-synpatic sites. Chronic blockade of LTCCs with nimodipine injection reversed NMDAR synaptic deficiency and impairment in spatial learning.

Consistent with previous evidence showing pretangle tau toxicity,^3,5,6^ the current study further demonstrates pretangle tau-induced synaptic and learning deficiency. A recent model that seeds htauE14 in the rat locus coeruleus to mimic the human origin of pretangle tau^13,16^ reported pretangle tau toxicity in the absence of NFT. Locus coeruleus htauE14-infused rats exhibit impairment in olfactory discrimination learning and spatial learning, correlating with a reduction of noradrenergic fibres in the olfactory cortex and hippocampal dentate gyrus.^13,16^ Olfactory deficiency is one of the earliest signs of preclinical Alzheimer’s disease.^21,22^ Locus coeruleus (LC) fibre degeneration and neuronal loss correlate with Braak stages^23,24^ and were observed in preclinical subjects.^25^ These observations suggest that pretangle tau indeed drives preclinical neuronal degeneration and behavioural changes. Furthermore, htau seeding without hyperphosphorylation in the rat LC in another study showed negligible effects of neuronal toxicity.^26^ Together, these reports and our current study suggest that the degree of abnormal tau phosphorylation appears to be a decisive factor in tau pathology. However, the model has limitations in that it does not mimic the natural temporal course of phosphorylation at various sites in tau pathology.

Hippocampal CA1 is particularly vulnerable to calcium dysregulation and tau pathology during aging and Alzheimer’s disease. In aging, excess calcium influx through LTCCs mediates the enlargement of medium and slow afterhyperpolarization.^19,27^ Augmentation of slow afterhyperpolarization in the aged CA1 is associated with reduced neuronal excitability and impaired hippocampus-dependent learning, which is reversed by nimodipine administration.^10,15,19^ LTCC hyperfunction associated neuronal hypoexcitability impairs NMDAR-dependent LTP,^28^ which may underlie cognitive decline during aging. Decreased tendency for LTP and increased tendency for LTD during aging are attributed to LTCC hyperfunction and concomitant reduced function of NMDARs.^29^ Additionally, a shift from NMDAR- to LTCC-dependent LTP has been reported at the CA1 synapses.^30^ Our finding of neuronal and synaptic changes induced by hyperphosphorylated tau resembles those reported in aging: decreased CA1 neuronal excitability and impaired NMDAR-LTP were paralleled by concomitant increased expression and function of LTCCs. These results suggest tau hyperphosphorylation accelerates senescence-associated cellular changes. Nimodipine administration reversed spatial learning deficiency by normalizing LTCC somatic membrane expression, neuronal excitability and NMDAR synaptic plasticity. In an aging study, nimodipine is more effective in reducing CA1 afterhyperpolarization and learning deficiency in aged animals,^19^ while in our study, nimodipine was effective in younger adult animals expressing hyperphosphorylated tau. It would be interesting in the future, to examine the expression levels of LTCC in aging rats in our model and test the effects of nimodipine in reversing later stage synaptic deficiency.

Interestingly, we observed a concomitant shift of NMDAR GluN1 subunit from synaptic to extra-synaptic sites, which could also directly affect NMDAR-dependent LTP. The shift in GluN1 trafficking was also prevented by nimodipine injection. In a P301S tau mouse model, hyperphosphorylated tau leads to a reduction of NMDARs via histone methyltransferase-mediated GluN1 ubiquitination.^31^ GluN1 downregulation was not observed in our rats with 1–3 months of pretangle tau incubation. Tau has been shown to mediate both synaptic^32^ and extra-synaptic^33^ NMDAR toxicity and may regulate NMDAR trafficking from synaptic to extra-synaptic sites through actin depolymerization.^33^ Our data suggest that reducing LTCC hyperfunction also normalizes NMDAR synaptic expressions.

Correlational links between tau hyperphosphorylation and LTCCs have been reported. Hippocampal neurons in Alzheimer’s disease patients show higher binding of isradipine, an LTCC dihydropyridine ligand and increased cell loss than non-dementia brains,^12^ suggesting selective vulnerability of the hippocampus to calcium dysregulation in Alzheimer’s disease. Similarly, increased level of intraneuronal calcium in the hippocampus of transgenic 3xTg Alzheimer’s disease mice has been attributed to LTCCs.^34^ A selective correlation of hyperphosphorylated tau in the CA1^35^ and LTCC hyperfunction^36^ in the 3xTg mice has been established. A recent study showed that specific human tau isoforms, such as 0N4R used in our study, enhance LTCC currents and associated medium and slow afterhyperpolarizations in cultured hippocampal neurons.^37^ Similar findings have been reported in *Drosophila* mushroom body, with 0N4R tau isoform increasing LTCC function and impairing olfactory learning.^38^ Our study showed more severe and immediate deficiency induced by hyperphosphorylated tau (htauE14) than human tau and subsequently focused on LTCC function and synaptic plasticity at a time point where behavioural impairment was observed in htauE14, but not htau rats. Consistent with more severe learning impairment, htauE14 resulted in a higher amount of tau phosphorylation at S262 and S356, two phosphorylation sites at the microtubule binding domain involved in early tau pathology.^39^

The molecular signalling involved in tau hyperphosphorylation-induced LTCC hyperfunction requires further investigation. A potential link between tau and LTCCs could be Src family kinases.^40^ Tau interacts with Src kinases and their activator Pyk2^41-43^ and could potentially enhance LTCC activation. In turn, LTCC activation mediates tau hyperphosphorylation *via* GSK3β, which can be prevented by an LTCC blocker *in vitro.*^44^ The interaction between hyperphosphorylated tau and LTCCs thus forms a positive feedback loop leading to intracellular calcium overload and associated neuronal toxicity in preclinical stages.

Restoring calcium homeostasis has been one of the therapeutic strategies in Alzheimer’s disease research. LTCC blockers have shown to be neuroprotective in animal models. For example, isradipine, an Food and Drug Administration-approved dihydropyridine LTCC blocker, lowered tau and Aβ burden and improved cognition in mouse models.^45^ In humans, evidence for the effect of LTCC blockers is controversial. Some beneficial results of nimodipine on cognition have been reported in patients with various types of dementia,^18,20^ while a large clinical trial only showed moderate benefits in secondary outcome measures.^46^ However, it should be noted that clinical trials targeted patients at late Braak stages, where neurological deficiency may be irreversible. Our research shows that LTCC hyperfunction may occur early in pretangle tau stages and early intervention strategies are likely more beneficial. Furthermore, the recent development of novel therapeutic agents that target LTCC as part of a multi-target approach has emerged as a potential effective therapeutic treatment.^44^ Alternatively, targeting pretangle tau pathology, serving both upstream and downstream of LTCC hyperfunction, is a viable strategy.

## Supplementary Material

fcae096_Supplementary_Data

## Data Availability

The authors confirm that the data supporting the findings of this study are available within the article and its supplementary material.
