# Supplementary material for: Hippocampal hyperphosphorylated tau-induced deficiency is rescued by L-type calcium channel blockade
Source: Brain Commun. 2024 Mar 20;6(2):fcae096. doi: 10.1093/braincomms/fcae096 (PMC10984573; doi:10.1093/braincomms/fcae096)
Supplement: fcae096_Supplementary_Data [file fcae096_supplementary_data.pdf]

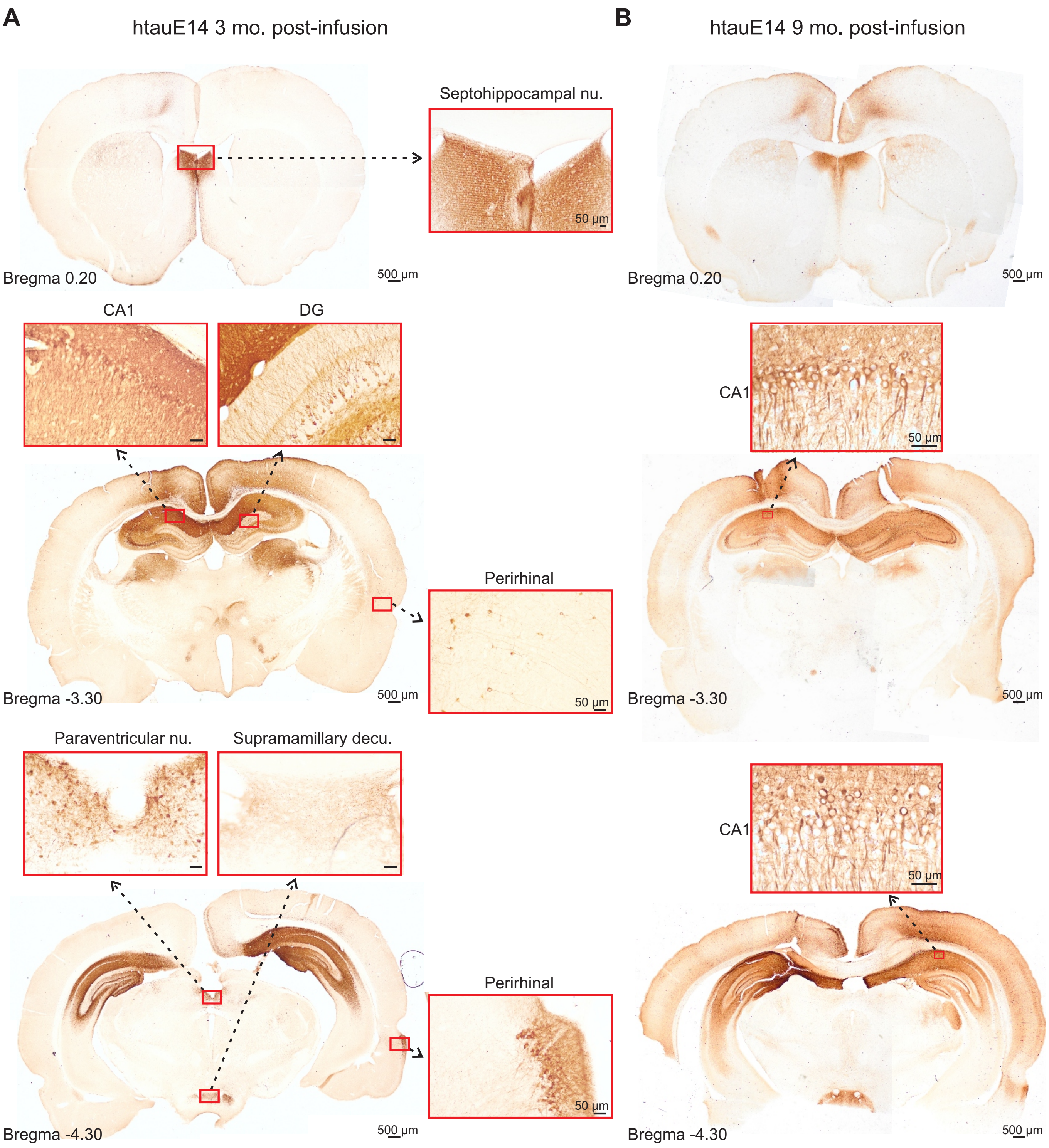

Supplementary Figure 1. Transductions of htauE14 in the brains 3 (**A**) or 9 months (**B**) post-infusion of AAVs show similar patterns. GFP^+ cells were observed in the hippocampus (CA1, DG) and projections such as the septohippocampal nuclei and supramamillary decussation. GFP^+ cells were also observed in the perirhinal cortex, likely due to transneuronal spread.

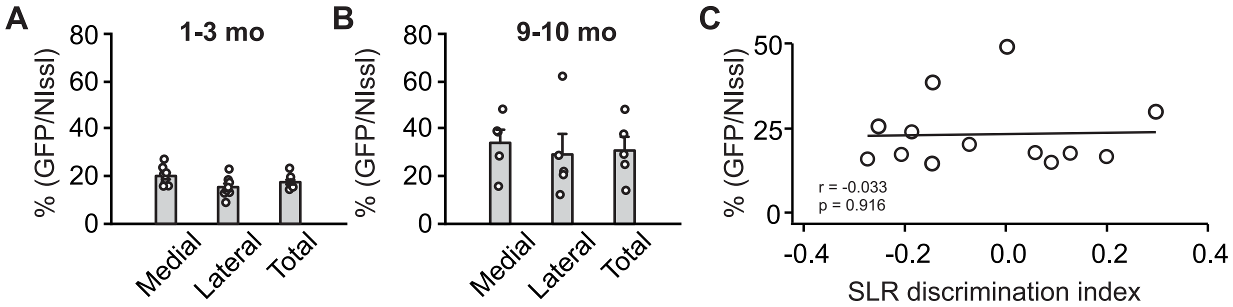

Supplementary Figure 2. No correlation of htauE14 transduction rate and spatial learning performance (SLR: spontaneous location recognition). (**A**) Transduction rate at 1-3 months indexed as %(GFP/Nissl). (**B**) Transduction rate at 9-10 months post-infusion. Note that transduction at 9-10 months has higher percentage over total cell counts than 1-3 months ($t = 2.889$, $P = 0.015$; t-test), suggesting either reduced tau clearance or potential transneuronal spread. (**C**) Pearson correlation between the transduction rate and SLR discrimination index. One-way ANOVAs were used for statistical comparisons in (A) and (B). Data points represent individual animals.

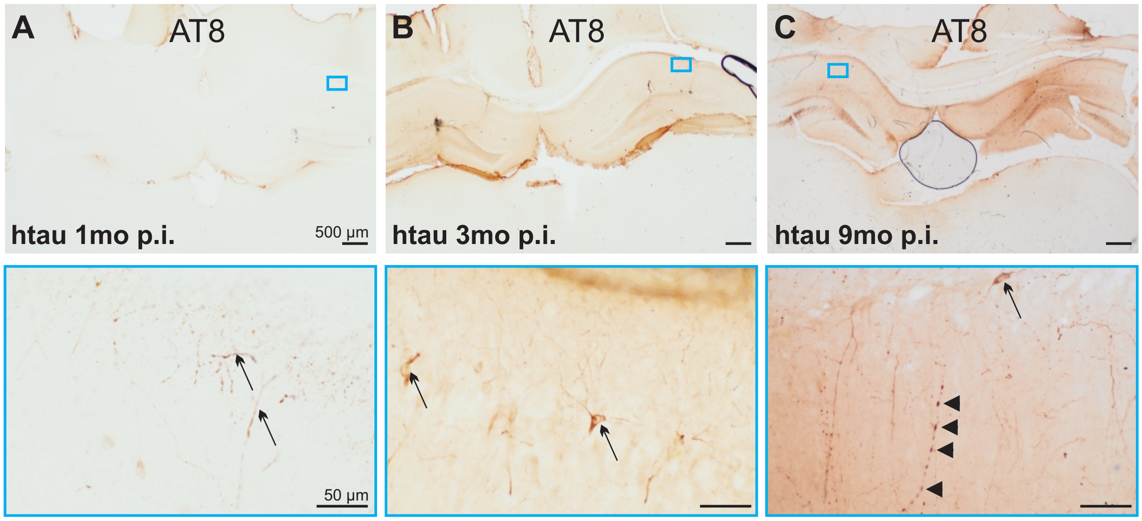

Supplementary Figure 3. Tau phosphorylation in htau hippocampus indexed by an antibody AT8 that recognizes S202/T205 phosphorylation sites. (**A-C**) AT8 staining in htau brains 1, 3, and 9 months post-infusion. At 9 months, the AT8 staining shows striation pattern in the processes (arrow heads). Arrows indicate $AT8^{+}$ staining (cells or processes). Lower panels are zoom in images in the blue boxes in the upper panels.

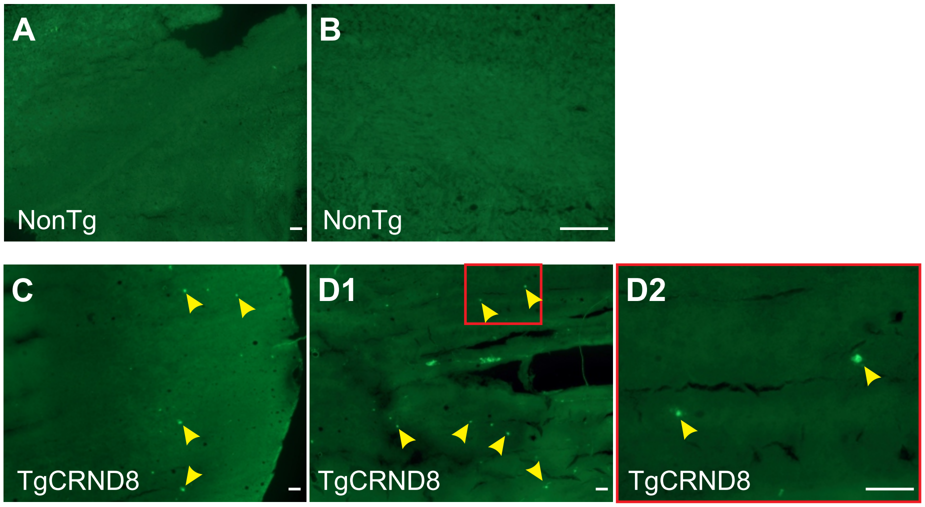

Supplementary Figure 4. Thioflavin S negative control in a WT mouse tissue (**A, B**) and positive control in a TgCRND mouse brain (**C, D1, D2**). D2 is the zoom in image of the area labeled by the red box in D1. Yellow arrowheads indicate example positive staining. Scale bars: 100 µm.

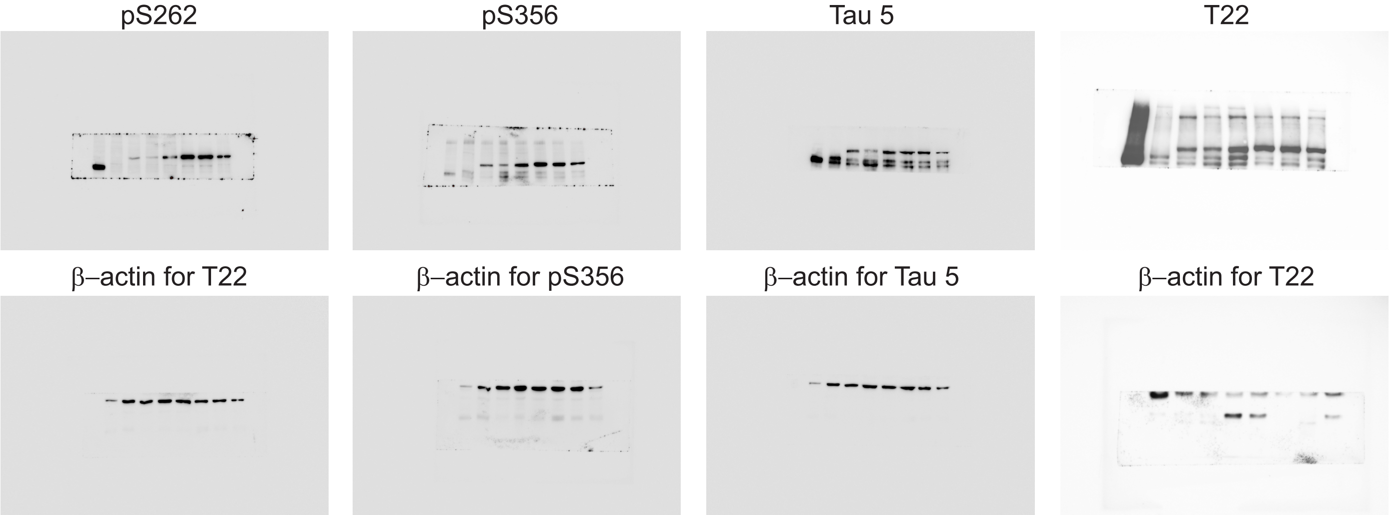

Supplementary Figure 5. Uncropped Western blots for Figure 2I-L. Note the blot membrane was cut into two for the probing of the specific antibody (top with higher MW) and β-actin (lower MW).

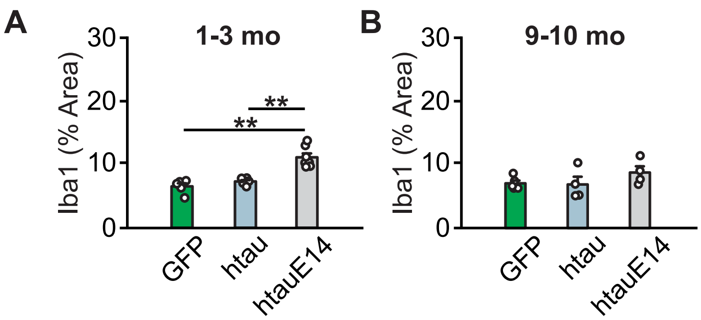

Supplementary Figure 6. Iba1 cell densitometry comparison among groups. Densitometry analysis were conducted in ImageJ. The percentage of Iba-1 positive area over the whole area of CA1 layer were computed. **(A)** Iba1 cell densitometry at 1-3 months post-infusion ($F(2,15)$ = 25.644, P <0.001; N = 5/6/7). **(F)** Iba1 cell densitometry at 9-10 months post-infusion ($F(2,10)$ = 1.326, P = 0.308; N = 5/4/4). **P<0.01. One-way ANOVAs were used for statistical analysis. Data points represent individual animals.

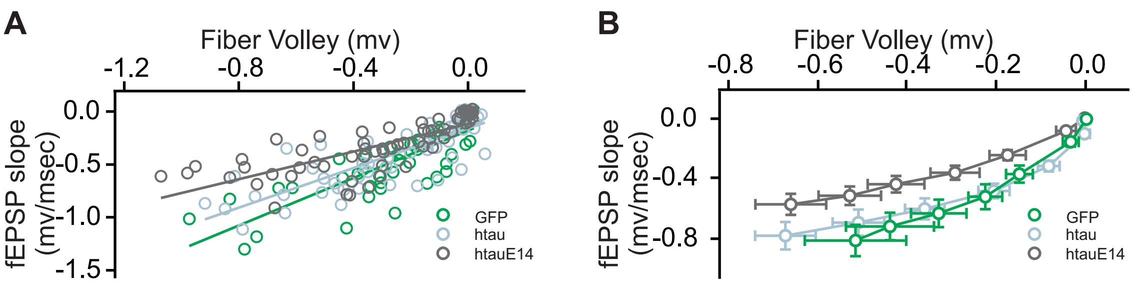

Supplementary Figure 7. Input-output relationship between presynaptic fiber volley and fEPSP slope in 1-3 months post-infusion rats. (**A**) Individual data points of FV against fEPSP slope. (**B**) Average input-output curves of the three groups. Error bars represent standard errors of the means.

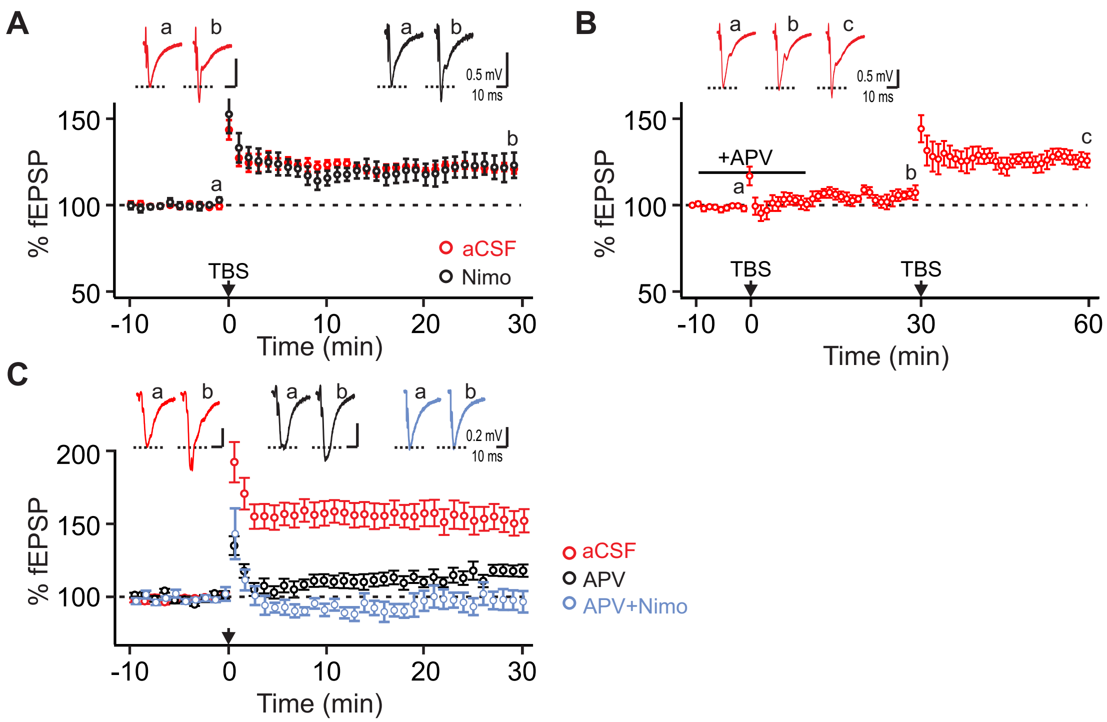

Supplementary Figure 8. Characterization of theta-burst stimulation (TBS) and 200 Hz LTP protocols. **(A)** TBS-induced LTP was not affected by nimodipine application during the induction. **(B)** TBS-induced LTP was prevented by NMDAR blockade with APV. LTP appeared following induction after APV wash. **(C)** 200 Hz protocol generated compound LTP consisting of an LTCC and an NMDAR component.

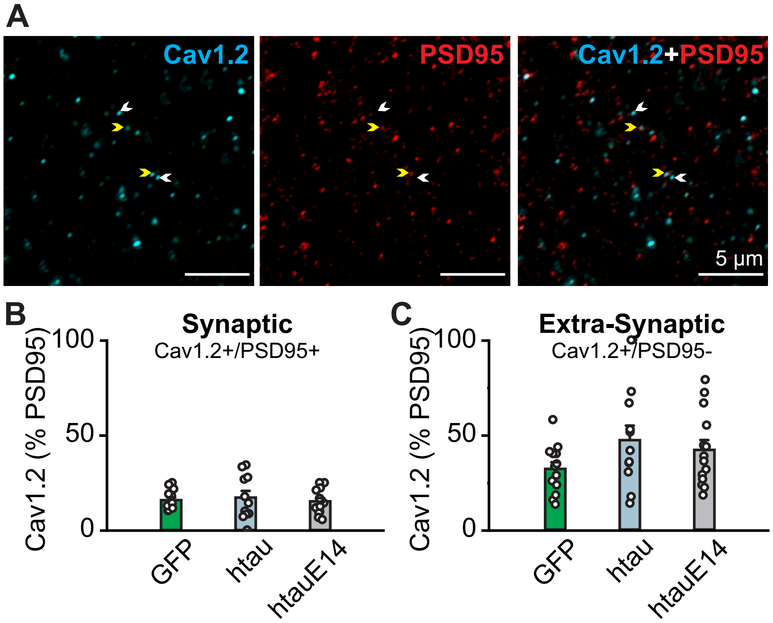

Supplementary Figure 9. LTCC Cav1.2 expression at synaptic and extra-synaptic sites were not different among GFP, htau and htauE14 hippocampal CA1 neurons. **(A)** Example images of Cav1.2 (blue) and PSD95 (red) staining. Yellow arrow heads show co-localization of Cav1.2 and PSD95. White arrow heads show Cav1.2 puncta not co-localized with PSD95. **(B)** Percentage of synaptic Cav1.2 puncta over PSD95 ($F(2,39) = 0.378$, $P = 0.688$). **(C)** Percentage of extra-synaptic Cav1.2 puncta over PSD95 ($F(2,39) = 1.640$, $P = 0.207$). Scale bars, 5 µm. One-way ANOVAs were used for statistical analysis. Data points represent individual slices.

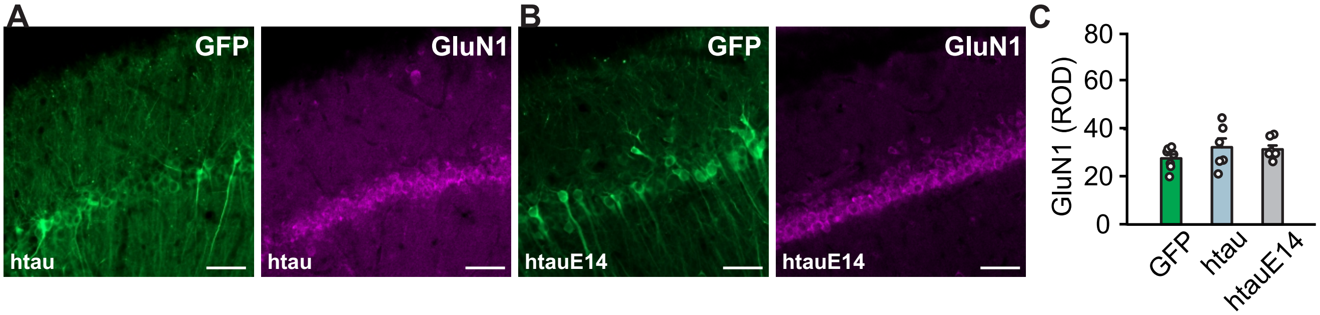

Supplementary Figure 10. NMDAR subunits GluN1 expressions in GFP, htau and htauE14 hippocampal CA1 neurons. (**A**) Example images of GluN1 staining in GFP-expressing CA1 cells in an htau animal. (**B**) Example images of GluN1 staining in GFP-expressing CA1 cells in an htauE14 animal. (**C**) Relative optical density (ROD) of GluN1 among three groups ($F(2,17) = 1.096$, $P = 0.357$). Scale bars, 50 µm. One-way ANOVA was used for statistical analysis. Data points represent individual slices.

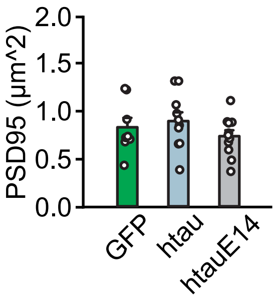

Supplementary Figure 11. PSD95 density in GFP, htau and htauE14 hippocampal CA1 neurons ($F(2,26) = 0.996$, $P = 0.383$). One-way ANOVA was used for statistical analysis. Data points represent individual slices.

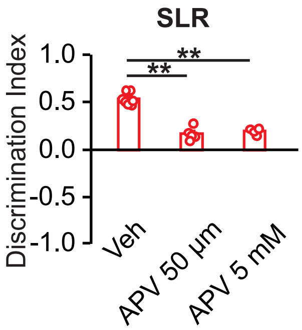

Supplementary Figure 12. Spontaneous location recognition (SLR) was dependent on hippocampal CA1 NMDAR ($F(2,13) = 69.178$, $P < 0.01$). **$P < 0.01$. One-way ANOVA was used for statistical analysis. Data points represent individual animals.

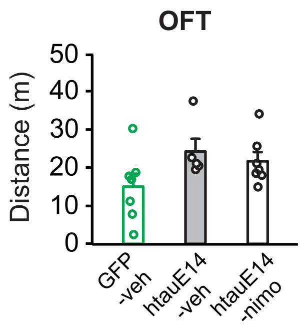

Supplementary Figure 13. Movement indexed by distance traveled in the open field test (OFT) was not affected by nimodipine chronic injections ($F(2,16) = 2.383$, $P = 0.124$). One-way ANOVA was used for statistical analysis. Data points represent individual animals.
